# Exotic helium compounds and new states under planetary conditions

**DOI:** 10.1093/nsr/nwaa121

**Published:** 2020-06-06

**Authors:** Erio Tosatti

**Affiliations:** International School for Advanced Studies (SISSA), Italy; CNR-IOM Democritos National Simulation Center, Italy; The Abdus Salam International Centre for Theoretical Physics (ICTP), Italy

The variety of states that matter may take under extreme conditions of pressure and temperature—some of which are being discovered by theoretical computer simulations as well as by real experiments—sometimes exceeds and defies fantasy. Multi-megabar pressure conditions, quite hard to realize in the laboratory, are expected to prevail in the core of planets. While these high-pressure states determine to some extent the planetary evolution, our physical understanding is by necessity largely dependent on theory. It is thus fortunate that computer simulations of the first-principles kind, whose accuracy increases with density, can be put to work, leading sometimes to real discoveries, such as that of superionic water [1]. A very fresh and intriguing discovery—because it involves helium, the most inert element in the universe—has just appeared.

The gaseous atmosphere of icy giant planets such as Uranus and Neptune is composed mostly of hydrogen and helium, while the inner mantle is mainly composed of water, ammonia and methane [2]. An open question is whether helium could penetrate into the mantle and react with molecular species found there giving rise to unheard of compounds that only exist under conditions of ultra-high pressure. Using first-principles simulations supplemented by machine-learning accelerated crystal structure prediction methods, Gao *et al*. [3] of Jian Sun's computational condensed matter group at Nanjing University investigated the possibility of stable helium-methane compounds. Amazingly, they predicted a He_3_CH_4_ compound that is stable over a wide range of pressures from 55 to 155 GPa.

The He_3_CH_4_ compound is predicted as a molecular crystal composed of helium atoms and methane molecules, which is a nice example of pure van der Waals crystals. The insertion of helium atoms changes the original packing of pure methane molecules and also largely hinders the polymerization of methane at higher pressures. At high temperatures, this unexpected compound has a phase transition from a regular solid (Fig. 1a) to a plastic phase (Fig. 1b), where methane molecules rotate freely, to a further phase with coexistence of diffusive helium and plastic methane (Fig. 1c). Superionic-like, but with diffusive neutral atoms rather than ions, this kind of phase has never been discovered before.

**Figure 1.**
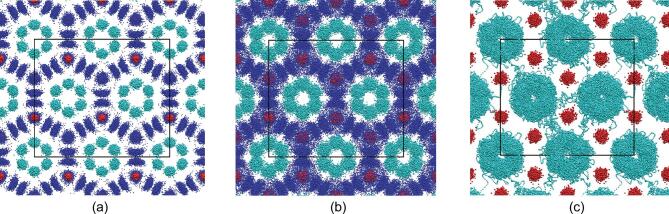
Trajectories of high pressure He_3_CH_4_ phases from *ab initio* molecular dynamics simulations at 50 GPa and 1000 K, 1900 K and 2350 K, respectively. (a–c) Blue, cyan and red dots represent H, He and C atoms, respectively. At 2350 K, the trajectories of H and He atoms overlap with one another, and therefore only the He and oxygen trajectories are shown here. (Figure is taken from [3].)

With similar methods, Jian Sun’s group also predicted helium-water [4] and helium-ammonia [5] compounds, including plastic and superionic phases under planetary conditions. Highly dependable, these density functional theory based predictions of chemically unlikely helium compounds will stimulate further experimental investigations, including shockwave compression used in the recent observations on superionic water [6]. These results actually suggest that current models of icy giant planets may need to be updated, to include previously unexpected helium compounds and states of matter in the model of planet interiors.

## FUNDING

This work was supported by ERC Advanced Grant N. 8344023 ULTRADISS.

***Conflict of interest statement*.** None declared.
